# Induction Therapy Followed by Surgery in Advanced Thymic Epithelial Tumors: A 20-Year Systematic Review and Meta-Analysis

**DOI:** 10.32604/or.2026.077158

**Published:** 2026-07-16

**Authors:** Giovanni Leuzzi, Michele Ferrari, Federica Sabia, Alessandro Pardolesi, Alessia Stanzi, Claudia Proto, Giuseppe Lo Russo, Arsela Prelaj, Monica Ganzinelli, Matteo Calderoni, Clarissa Uslenghi, Ugo Pastorino, Piergiorgio Solli

**Affiliations:** 1 Division of Thoracic Surgery, Fondazione IRCCS Istituto Nazionale dei Tumori, Milan, Italy; 2 Department of Medical Oncology, Fondazione IRCCS Istituto Nazionale dei Tumori, Milan, Italy; 3 Lung Cancer Prevention Research, Fondazione IRCCS Istituto Nazionale dei Tumori, Milan, Italy

**Keywords:** Induction therapy, thymic epithelial tumors, metaregression, survival, CAP, multimodality therapy

## Abstract

**Backgrounds:** Despite the availability of multimodal strategies, no universally accepted guidelines exist for the management of advanced Thymic Epithelial Tumors (TETs), particularly in locally advanced thymomas. The aim of this study was to evaluate the oncological and surgical outcomes of induction therapy (IT) followed by surgery in patients with Masaoka–Koga stage III–IVA TETs. To this end, we conducted a systematic review and meta-analysis assessing surgical-pathological and survival outcomes. **Methods:** Following Preferred Reporting Items for Systematic Reviews and Meta-Analyses (PRISMA) guidelines, a systematic search of PubMed, Embase, and the Cochrane Central Register of Controlled Trials was performed. Twenty-four studies published between 2003 and 2023 were included, comprising 749 patients treated with IT before surgical resection. The co-primary endpoints were Overall survival (OS) and Progression-free survival (PFS). Random-effects meta-analysis assessed pooled outcomes, while heterogeneity, publication bias, and meta-regression analyses were performed to explore potential moderators (year, histology, stage). The study was registered in Prospective Register of Systematic Reviews (PROSPERO) (CRD420251026044). **Results:** Of the included studies, 6 were prospective and 18 retrospective; 9 analyzed thymomas only, while 15 included both thymomas and thymic carcinomas. Regarding stage distribution, 4 studies focused on stage III, 11 on stage III–IV, 3 on stage IV, and 2 also included earlier stages. Response to IT was assessed by Response Evaluation Criteria in Solid Tumors (RECIST) in 11 studies and World Health Organization (WHO) criteria in 4. The pooled rate of radiological response to IT, completeness of resection, 5-year OS, 10-year OS and 5-year PFS were 62.8%, 71.6%, 77.6%, 54.3% and 55.6%, respectively. Meta-regression showed histology significantly influenced 10-year OS (*p*-value 0.0418) as well as on PFS (*p*-value 0.0042) and treatment period on PFS (*p*-value 0.0007). **Conclusions:** Induction therapy followed by surgery provides acceptable long-term outcomes in advanced TETs. Histology remains a key prognostic factor, but 10-year OS has not improved over the past two decades, underscoring the need for innovative, histology-tailored therapeutic strategies to enhance survival.

## 1 Introduction

Thymic epithelial tumors (TETs) are rare malignancies and represent the most common neoplasms of the anterior mediastinum in adults. The incidence of these tumors is estimated to be between one and five cases per million people per year [1]. Because of their rarity, most available evidence derives from retrospective single-center experiences, limiting the development of standardized treatment algorithms and high-level evidence-based recommendations.

Thymic malignancies encompass a spectrum of diseases, including thymomas and thymic carcinomas, which differ in their biological behavior and prognosis. Compared to thymomas, thymic carcinomas are particularly aggressive, with a higher likelihood of locoregional invasion and distant metastasis at diagnosis, leading to poorer survival outcomes [1,2].

Early-stage TETs (stage I–II) can usually be managed with surgery alone, which remains the cornerstone of treatment. However, a considerable proportion of patients present with locally advanced disease (Masaoka-Koga stage III–IVA) in which a complete resection is more complex but remains a key factor to improve long-term survival and reduce recurrence rates [3,4,5]. Due to the aggressive behavior of advanced thymic tumors (ATTs) and their propensity to infiltrate adjacent vital structures, achieving a radical resection is often unfeasible at the time of diagnosis. In such cases, induction therapy (IT) is frequently employed in order to downstage the tumor and enhance resectability in experienced hands. Specifically, cisplatin-based chemotherapy, with or without radiotherapy, has been widely used in this setting and has demonstrated highly-variable response rates (between 22% and 92%) [6] and good tolerability in several clinical studies [7,8,9]. On the other hand, combination of radiotherapy into induction protocols continues to be an area of active research, with some studies indicating enhanced tumor control but no clear survival benefit [10,11,12].

While multimodal treatment strategies are available, pre-operative chemotherapy is more strongly indicated for thymic carcinomas than for locally-advanced thymomas, which currently lack universally accepted management guidelines [12]. As well, the rarity of TETs along with the scarcity of high-quality prospective studies have resulted in significant variability in treatment strategies across various Institutions. These data highlight the need for a thorough assessment of neoadjuvant strategies to help surgeons and oncologist in clinical decision-making. The objective of this study was to systematically evaluate the outcomes of IT followed by surgery in patients with stage III–IVA TETs. To this end, we conducted a systemic review and meta-analysis to evaluate overall survival (OS) and progression-free survival (PFS) as co-primary endpoints, as well as surgical, pathological and oncological outcomes in patients with stage III–IVA TETs who underwent IT followed by surgical resection. Specifically, we aimed to assess whether IT followed by surgery is associated with favorable survival outcomes in this setting.

## 2 Methods

### 2.1 Search Strategy and Study Selection

This meta-analysis is registered in the International Prospective Register of Systemic reviews (PROSPERO ID: CRD420251026044). The Preferred Reporting Items for Systematic Reviews and Meta-Analyses (PRISMA) 2020 guidelines were used to perform the systematic review and meta-analysis. The PRISMA 2020 checklist is provided as Supplementary Material.

On 20 February 2025, we conducted a comprehensive literature search using PubMed (United States National Library of Medicine), Embase, and the Cochrane Central Register of Controlled Trials (CENTRAL) to identify original studies published in English. The search included studies published between January 2003 and December 2023, corresponding to a 20-year study period.

Our search strategy incorporated relevant keywords and corresponding MeSH terms: “Surgery”, “survival”, AND (“thymus neoplasm” OR “thymoma” OR “thymic carcinoma”) AND (“neoadjuvant” OR “induction therapy”).

A qualified medical librarian (see Acknowledgements) reviewed the search strategy. The detailed search strategies for each database are provided in Appendix A. Two independent investigators (MF and GL) screened and selected all eligible studies. The selection process began with title screening, followed by abstract evaluation and, finally, with full text analyses for final inclusion into meta-analysis. In cases of disagreement, a third reviewer (PGS) was consulted to ensure consistency and resolve discrepancies.

Inclusion criteria were: (1) study enrolling 10 or more patients on Masaoka-Koga stage III-IVa thymoma or thymic carcinoma proven by pathology; (2) provided information on IT; (3) provided information on response rates; (4) provided information on survival; (5) inclusion of the most recent publication data repeatedly reported by the same author. Exclusion criteria were: (1) letters, case reports, or editorials; (2) abstracts or unpublished studies; (3) non-English articles; (4) studies on patients undergoing debulking or palliative resections or tumor biopsies; (5) animal research; (6) laboratory studies; (7) studies lacking the required data as specified in the inclusion criteria. To supplement the database search, we manually reviewed the reference lists of all retrieved articles and cross-checked relevant review papers. Review articles were also evaluated for discussion purpose. Weakness of design or data quality were not considered as exclusion criteria.

Study quality was assessed using the Newcastle–Ottawa Scale (NOS) for cohort studies, evaluating three domains: selection of study groups, comparability of cohorts, and outcome assessment. Each study was assigned a score ranging from 0 to 9 points.

The collected data included: first author, year of publication, country, study design, number of institutions involved, recruitment period, sample size, patient demographics, Masaoka staging, WHO histological classification, biopsy methods, IT protocols, surgical techniques, completing planned IT, adjuvant treatments, radiological response (RR) to IT, complete resection rate, follow-up duration, progression-free survival (PFS) outcomes, and overall survival (OS).

### 2.2 Summary Measures

Co-primary outcomes for the meta-analysis were overall survival (OS) and progression-free survival (PFS). Secondary outcomes included: (a) RR rate to IT, quantified as the sum of partial response (PR) and complete response (CR) on the total numbers of treated patients; (b) complete resection rate; (c) 5-year and (d) 10-year OS from the time of beginning IT or from the time of surgical resection as reported; and 5-year PFS. OS and PFS were estimated by the Kaplan-Meier method and the corresponding standard errors (SEs) were calculated [13]. When available, survival estimates were extracted from studies reporting outcomes after surgical resection to improve comparability across series. To improve comparability across studies and partially address heterogeneity related to time origin, the main pooled analyses and meta-regression were restricted to studies reporting survival from the time of surgery.

### 2.3 Statistical Analysis

Meta-analysis was performed using random effect summaries. Heterogeneity between studies was assessed by heterogeneity variance τ^2^ and the I^2^ statistics [14]: I^2^ 25% low, 50% moderate, and 75% substantial heterogeneity. Meta-analyses results were visualized through forest plots. Publication bias was investigated through funnel plot and the plot asymmetry was tested by Egger’s regression test [15].

Meta-regression was used to examine moderators of the effect, including year of publication, histology (thymoma only or thymona and thymic carcinoma) and stage (stage III cases only or both stage III and stage IV). Meta-regression results were visualized by bubble plots. Pre-specified subgroup analyses were performed to explore potential sources of heterogeneity according to histology (thymoma only vs. studies including thymic carcinoma). Additional exploratory pooled analyses were performed for studies reporting survival outcomes in patients treated with upfront surgery to provide descriptive estimates. Separate pooled estimates were calculated when sufficient data were available.

All analysis were performed using Software R version 4.4.3.

## 3 Results

### 3.1 Study Selection and Characteristics

**Figure 1:**
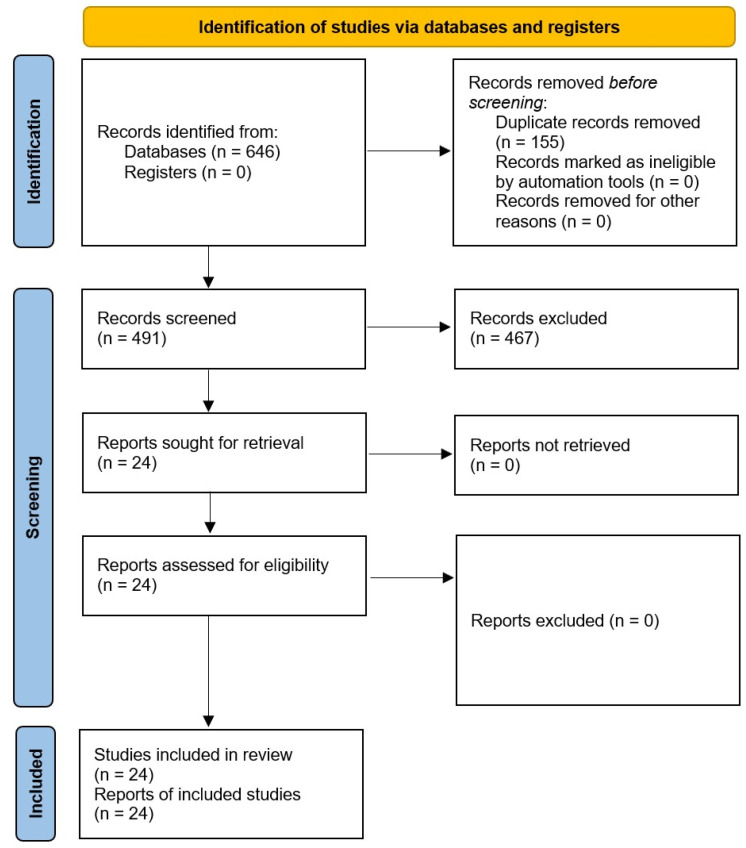
Flowchart of literature search and study selection.

A total of 646 records were identified through database searching. After the removal of 155 duplicates, 491 records were screened. Of these, 467 were excluded based on title and abstract screening. Twenty-four full-text articles were assessed for eligibility. Twenty-four studies were ultimately included in the qualitative and quantitative synthesis [7,9,10,16,17,18,19,20,21,22,23,24,25,26,27,28,29,30,31,32,33,34,35,36]. The study selection process is summarized in the PRISMA 2020 flow diagram (Fig. 1).

Study characteristics and eligibility criteria are summarized in Table 1. 

**Table 1:** Characteristics of the trials included in the meta-analysis.

Study	Country	Deriod	Design	Elegibility	N	Age	Male (%)	Biopsy Procedures (N/%)	Histology (WHO)	Initial Stage MASAOKA
Venuta, 2003 [16]	Italy	1989–2003	prospective	unresectable III	15	NA	NA	anterior mediastinotomy 7 (46.7), VATS 8 (53.3)	Thymic epitelial tumor	III: 15 (100)
Kim, 2004 [9]	USA	1990–2000	prospective	unresectable III, IVA–IVB	22	median 47 (25–70)	40.9	NA	Thymic epitelial tumor	III: 11 (50), IVA: 10 (45.5), IVB: 1 (4.5)
Lucchi, 2006 [20]	Italy	1989–2004	prospective	III–IVA	30	mean 53.7 (25–74)	43.3	anterior mediastinotomy 5 (16.7), VATS 7 (23.3), FNAB 4 (13.3), none 14 (46.7)	AB: 3 (10), B1: 5 (16.7), B2: 7 (23.3), B3: 15 (50)	III: 20 (66.7), IV: 10 (33.3)
Huang, 2007 [19]	USA	1996–2006	retrospective	IVA	18	median 43.5 (26–74)	44.4	NA	AB: 1 (5.6), B1: 2 (11.1), B2: 3 (16.7), B3: 6 (33.3), a mixture of B subtypes: 6 (33.3)	IVA: 18 (100)
Wright, 2008 [26]	USA	1997–2006	retrospective	unresectable III–IVA	10	median 53.5 (34–66)	30	NA	A: 1 (10), B1: 1 (10), B3: 7 (70), thymic carcinoma: 1 (10)	III: 7 (70), IVA: 3 (30)
Mineo, 2010 [17]	Italy	1989–2008	retrospective	unresectable III	33	Mean 55.5 ± 7.3	60.6	Anterior mediastinotomy 12 (36.4), VATS:21 (63.6)	A: 5 (15.2), AB: 6 (18.2), B1: 5 (15.2), B2: 10 (33.3), B3: 7 (21.2)	III: 33 (100)
Cardillo, 2010 [25]	Italy	1991–2007	retrospective	unresectable III–IVA	31	Mean 45.7 ± 12.5	77.4	Anterior mediastinotomy 31 (100)	AB: 8 (25.8), B1: 3 (9.7), B2: 4 (12.9), B3: 6 (19.4), thymic carcinoma: 10 (32.3)	III: 18 (58.1), IVA: 13 (41.9)
Kunitoh, 2010 [7]	Japan	1997–2005	prospective	unresectable III	11	NA	NA	NA	23 Tymoma	III: 21 (100)
Rena, 2011 [18]	Italy	1998–2008	retrospective	IVA (pleural implants)	18	mean 54.5 (29–68)	55.6	anterior mediastinotomy: 16 (88.9), FNAB:2 (11.1)	AB: 1 (5.6), B1: 2 (11.1), B2: 4 (22.2), B3: 7 (38.9), a mix of B subtypes: 4 (22.2)	IVA: 18 (100)
Rea, 2011 [24]	Italy	1980–2008	retrospective	III, IVA–IVB	38	NA	NA	NA	A: 3 (7.9), AB: 5 (13.2), B1: 4 (10.5), B2: 12 (31.6), B3: 8 (21.1), thymic carcinoma: 6 (15.8)	III: 23 (60.5), IVA: 12 (31.6), IVB: 3 (7.9)
Park, 2013 [23]	korea	2007–2011	prospective	III–IV	27	median 54 (15–68)	59.3	NA	B2: 4 (14.8), B3: 3 (11.1), C: 18 (66.7), a mix of B subtypes: 2 (7.4)	III: 8 (26.3), IVA: 17 (63.2), IVB: 2 (10.5)
Filosso, 2013 [28]	Italy	2000–2011	retrospective		11	NA	NA	5 anterior mediastinotomies, 5 VATS and 1 FNAB	Thymic carcinoma 11 (100)	NA
Korst, 2014 [10]	USA	2007–2012	prospective	III, IV, and I and II (only if >5 cm)	22	median 51 (18–78)	80	NA	A: 2 (10), AB: 1 (5), B1: 1 (5), B2: 3 (14), B3: 6 (29), C: 7 (33)	NA
Shintani, 2014 [34]	Japan	1998–2014	retrospective	III–V	16	Mean 52 ± 12	69	NA	Thymic carcinoma 13, neuroendocrine 2, unspecified 1	III: 11 (69), Ivb: 5 (31)
Cardillo, 2015 [27]	Italy	1990–2010	retrospective	III	108	Mean 51.5 ± 14.6	56	FNAB 51 (47), anterior mediastinotomy 37 (34), video-thoracoscopy 12 (11), mediastinoscopy 6 (6), antero-lateral minithoracotomy 2 (2)	A: 6 (6), AB: 18 (17), B1: 15 (14), B2: 26 (24), B3: 23 (21), C: 20 (19)	III: 108 (100)
Leuzzi, 2015 [31]	Italy	2001–2013	retrospective	III–IV	11	median 55	64	NA	A/AB/B1: 1 (9), B2/B3/C/NETT: 10 (91)	III: 4 (36), IVA: 5 (46), IVB: 2 (18)
Wei, 2016 [36]	China	1994–2012	retrospective	clinically stage III–IV	68	44.8 ± 14.9	63.2	NA	A: 2 (2.95), AB: 5 (7.4%), B1: 5 (7.4), B2: 8 (11.8), B3: 12 (17.6), thymic carcinomas: 34 (50), carcinoids: 2 (2.9)	NA
Kaba, 2018 [30]	Turkey	2002–2015	retrospective	IVa	25	NA	NA	NA	B1: 8 (20.5) B2: 16 (41) B3: 6 (15.4) thymic carcinoma: 9 (23)	NA
Park, 2018 [33]	Korea	2000–2013	retrospective	NA	110	50 ± 13	58.2	NA	Tymoma: 51 (50%) thymic carcinoma: 51 (50%)	I: 3 (3), II: 5 (5), III: 38 (35), IV: 64 (58)
Ma, 2019 [32]	Taiwan	2005–2013	retrospective	NA	45	59 (22–79)	47	NA	thymoma: 15 (33), A: (182), AB: 1 (2), B1: (182), B2: 1 (2), B2/B3: 1 (2), B3: (6) (13), not defined: 4 (9); thymic carcinoma: 30 (67)	III: 15 (33), IVA: 13 (29), IVB: 17 (38)
Nakamura, 2019 [22]	Japan	2003–2017	retrospective	NA	19	49 (32–70)	58	NA	B1 (*n* = 1), B2 (*n* = 13), and B3 (*n* = 5)	IV: 13 (68), 6 recurrent cases of pleural disseminations after surgical treatment
Suh, 2019 [35]	Korea	2000–2013	retrospective	III–IV	18	48.28 ± 11.68	61.1	NA	B1: 2 (11.1), B2: 4 (22.2), B3: 4 (22.2), C8 (44.4)	III: 13 (72), IVA: 3 (17), IVB: 2 (11)
Guan, 2023 [29]	China	2008–2019	retrospective	III–IV	31	NA	48	NA	B1: 4 (13), B2: 7 (23), B3: 5 (16), Thymic carcinoma: 15 (48)	III: 20 (65), IVB: 11 (35)
Abdel Jalil, 2023 [21]	jordan	2015–2021	retrospective	NA	12	NA	75	NA	AB: 2 (17), B2: 4 (33), B3: 6 (50)	I: 1 (8), III: 2 (16), IVA: 9 (39)

Note: WHO: World Health Organization; NA: not applicable.

Included studies were published from 2003 to 2023, 6 were prospective and 18 retrospective. In the considered studies, between 10 and 110 patients were included, with a total of 749 patients and a percentage of males ranging from 30 to 80%. Masaoka stage and WHO histology information were extracted. Nine studies included only thymomas [7,9,16,17,18,19,20,21,22], while the other 15 included both thymoma and thymic carcinoma [10,23,24,25,26,27,28,29,30,31,32,33,34,35,36]. Four, 11 and 3 studies included, respectively, only stage III tumors [7,16,17,27], stage III and stage IV TETs [9,20,23,24,25,26,31,32,34,35] and stage IV only [18,19,22]; 2 papers analyzed stage I and stage II tumors as well [21,23].

### 3.2 Meta-Analysis of Radiological Response to Induction Therapy and Completeness of Resection

Rates of RR to IT and completeness of resection are summarized in Table 2. 

**Table 2:** Kind of induction therapy, radiological response and completeness of resection.

Study	N	Kind of Induction Therapy	Radiological Response	Radiological Response Criteria	Complete Resection	Surgical Approach (%)
Venuta, 2003 [16]	15	CAP	CR (2); PR (8)	WHO	NA	Median sternotomy (15)
Kim, 2004 [9]	22	CAP	CR (3); PR (14)	WHO	17	NA
Lucchi, 2006 [20]	30	EEP	CR (2); PR (20)	NA	23	Median sternotomy: 26 (86.6), sternotomy and lateral thoracotomy: 4 (13.3)
Huang, 2007 [19]	18	CAP	CR (0); PR (12)	RECIST	12	Hemiclamshell: 8 (44.4), clamshell:2 (11.1), thoracotomy: 3 (16.7), sternotomy and lateral thoracotomy: 3 (16.7), sternotomy only: 1 (5.6), clamshell only: 1 (5.6)
Wright, 2008 [26]	10	EP + RT	CR (0); PR (4)	RECIST	8	NA
Mineo, 2010 [17]	33	EP	CR (12); PR (21)	NA	17	Sternotomy: 24 (72.7), combined: 9 (27.3)
Cardillo, 2010 [25]	31	CAP	CR (2); PR (16)	RECIST	NA	NA
Kunitoh, 2010 [7]	11	CODE	NA	WHO	9	NA
Rena, 2011 [18]	18	EP/ADOC	CR (1); PR (11)	RECIST	12	Hemiclamshell: 11 (61.1), thoracotomy: 2 (11.1), sternotomy plus lateral thoracotomy: 2 (11.1)
Rea, 2011 [24]	38	ADOC	CR (0); PR (26)	WHO	28	NA
Park, 2013 [23]	19	TP	CR (0); PR (10)	RECIST	15	NA
Filosso, 2013 [28]	11		CR (0); PR (9)	RECIST	9	NA
Korst, 2014 [10]	21	EP + RT	CR (0); PR (10)	RECIST	17	Median sternotomy: 19 (90.5), hemiclamshell: 1 (4.8), and clamshell: 1 (4.8)
Shintani, 2014 [34]	16	TP, ADOC, PC, EP, CODE	CR (0); PR (9)	NA	11	NA
Cardillo, 2015 [27]	108	ADOC, CAP, CEE + RT (5 pts)	NA	RECIST	81	median sternotomy: 96 (88.89), lateral thoracotomy: 8 (7.40), sternotomy and lateral thoracotomy: 4 (3.70).
Leuzzi, 2015 [31]	11	CAP	CR (0); PR (9)	WHO	9	NA
Wei, 2016 [36]	68	CAP, EP, PC	NA	NA	46	NA
Kaba, 2018 [30]	25	Platinum + RT	NA	NA	NA	NA
Park, 2018 [33]	110	CAP, ADOC, EP, PC, others	CR (3); PR (64)	RECIST	71	NA
Ma, 2019 [32]	45	NA	NA	NA	NA	NA
Nakamura, 2019 [22]	19	CAMP	CR (0); PR (15)	RECIST	NA	NA
Suh, 2019 [35]	18	CAP, ADOC + RT (5 pts)	CR (0); PR (13)	NA	13	NA
Guan, 2023 [29]	31	CAP, DP, TP + RT	CR (6); PR (16)	RECIST	23	NA
Abdel Jalil, 2023 [21]	23	CAP	CR (0); PR (1)	RECIST	NA	median sternotomy or thoracotomy 12 (52), VATS 11 (48)

Note: CR: complete response; PR: partial response; CAP: Cyclophosphamide, Adriamycin/Doxorubicin, Cisplatin; EEP: Etoposide, Epidoxorubicin, Cisplatin; EP: Etoposide, Cisplatin; CODE: Cisplatin, Vincristin, Doxorubicin, Etoposide; ADOC: Doxorubicin, Cisplatin, Vincristine, Cyclophosphamide; TP: Taxotere, Platinum; PC: Paclitaxel, Carboplatin; CEE: Cisplatin, Etoposide, Epirubicin; CAMP: Cyclophosphamide, Adriamycin/Doxorubicin, Methotrexate, Procarbazine; DP: docetaxel, cisplatin; VATS: video-assisted thoracic surgery; NA: not applicable.

Nineteen studies reported the rate of response to IT, CR and PR. The response was evaluated by RECIST (Response Evaluation Criteria In Solid Tumors) criteria in 11 studies, by WHO criteria in 4 studies, and without a description in 4 studies. The latter were excluded from the final analysis. 

**Figure 2:**
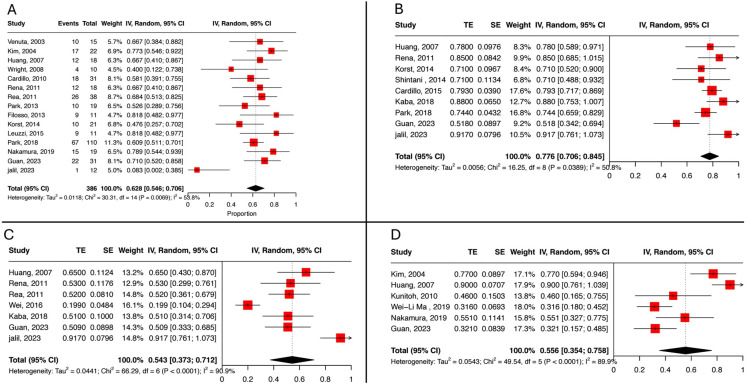
**Forest plots of response rate and survival.** (**A**) Forest plot and pooled rate of response rate to induction therapy according to standardized radiological response criteria (RECIST and WHO). (**B**) Forest plot and pooled rate of 5-year OS from the time of surgery. (**C**) Forest plot and pooled rate of 10-year OS from the time of surgery. (**D**) Forest plot and pooled rate of 5-year PFS. Arrows indicate confidence intervals extending beyond the limits of the graphical display. OS: overall survival; PFS: progression-free survival; RECIST: Response Evaluation Criteria in Solid Tumors; WHO: World Health Organization response criteria; CI: confidence interval; TE: treatment effect; SE: standard error; IV: inverse variance method; Tau^2^: between-study variance; Chi^2^: Cochran’s Q statistic; I^2^: inconsistency index.

Results of meta-analysis are shown in Fig. 2.

The pooled rate of RR to IT was 66.8% (95% confidence interval [CI], 57.3%–75.7%) with a substantial heterogeneity I^2^ 75.2%. Considering the subset of studies employing standardized RR criteria (RECIST and WHO) the pooled rate of response was 62.8% (95% confidence interval [CI], 54.6%–74.6%), with a moderate heterogeneity I^2^ 53.8% (Fig. 2A).

Twelve studies described the surgical approach while 18 reported information on completeness of resection after induction therapy. The pooled rate of complete of resection was 71.6% (95% CI, 67.7%–75.3%), with a low heterogeneity I^2^ 0%.

### 3.3 Co-Primary Outcomes: Overall Survival and Progression-Free Survival

Data of OS and PFS are reported in Table 3.

**Table 3:** 5-year and 10-year overall survival (OS) and 5-year progression free survival (PFS) among the selected studies.

Study	5-Year OS%	Time 5-Year OS	10-Year OS%	Time 10-Year OS	5-Year PFS%	Follow-Up (Months)
Venuta, 2003 [16]			90	from induction therapy		
Kim, 2004 [9]	95.0	from induction therapy			77	median 50.3
Lucchi, 2006 [20]	82.5	from induction therapy	82.5	from induction therapy		median 94
Huang, 2007 [19]	78.0	from surgery	65	from surgery	90	Median (range) 32.2 (1.4–129.9)
Wright, 2008 [26]	69.0	from induction therapy				median 41
Mineo, 2010 [17]	37.0	from induction therapy	24	from induction therapy		
Cardillo, 2010 [25]			57.9	from induction therapy		
Kunitoh, 2010 [7]	91.0	from induction therapy			46	
Rena, 2011 [18]	85.0	from surgery	53	from surgery		mean ± SD 82 ±33
Rea, 2011 [24]			52	from surgery		
Park, 2013 [23]	79.4 (4 years OS)	from surgery			40.6 (4 years PFS)	median 42.6
Filosso, 2013 [28]	75.0	from surgery	58	from surgery		
Korst, 2014 [10]	71.0	from surgery				median (range) 27 (0–64)
Shintani, 2014 [34]	71.0	from surgery				median 72
Cardillo, 2015 [27]	79.3	from surgery				median 71
Leuzzi, 2015 [31]	71.4 (3 years OS)	from surgery				
Wei, 2016 [36]	49.7	from induction therapy	19.9	from surgery		
Kaba, 2018 [30]	88.0	from surgery	51	from surgery		
Park, 2018 [33]	74.4	from surgery				
Ma, 2019 [32]	76.4	from induction therapy			31.6	
Nakamura, 2019 [22]	76.7	from induction therapy	76.7	from induction therapy	55.1	median (range) 59.7 (16.1–184.2)
Suh, 2019 [35]	69.1	from induction therapy				median (range) 46.8 (3.6–85.2)
Guan, 2023 [29]	51.8	from surgery	50.9	from surgery	32.1	
Abdel Jalil, 2023 [21]	91.7	from surgery	91.7	from surgery		mean 43.8

Nine studies reported 5-year OS from the time of surgical resection. The pooled 5-year OS was 77.6% (95% CI, 70.6%–84.5%), with a moderate heterogeneity I^2^ 50.8% (Fig. 2B). Nine studies reported 5-year OS from the time of the beginning of IT. The pooled 5-year OS was 72.1% (95% CI, 59.3%–85%), with a substantial heterogeneity I^2^ 87%. Seven studies reported 10-year OS from the time of surgical resection. The pooled 10-year OS was 54.3% (95% CI 37.3%–71.2%), with a substantial heterogeneity I^2^ 90.9% (Fig. 2C). Five studies reported 10-year OS from the time of the beginning of IT. The pooled 10-year OS was 66.2% (95% CI 42.7%–89.6%), with a substantial heterogeneity I^2^ 92%. Finally, 6 studies only reported 5-year PFS, with a pooled 5-year PFS of 55.6% (95% CI 35.4%–75.8%), and a substantial heterogeneity I^2^ 89.9% (Fig. 2D).

### 3.4 Meta-Regression Analysis

**Figure 3:**
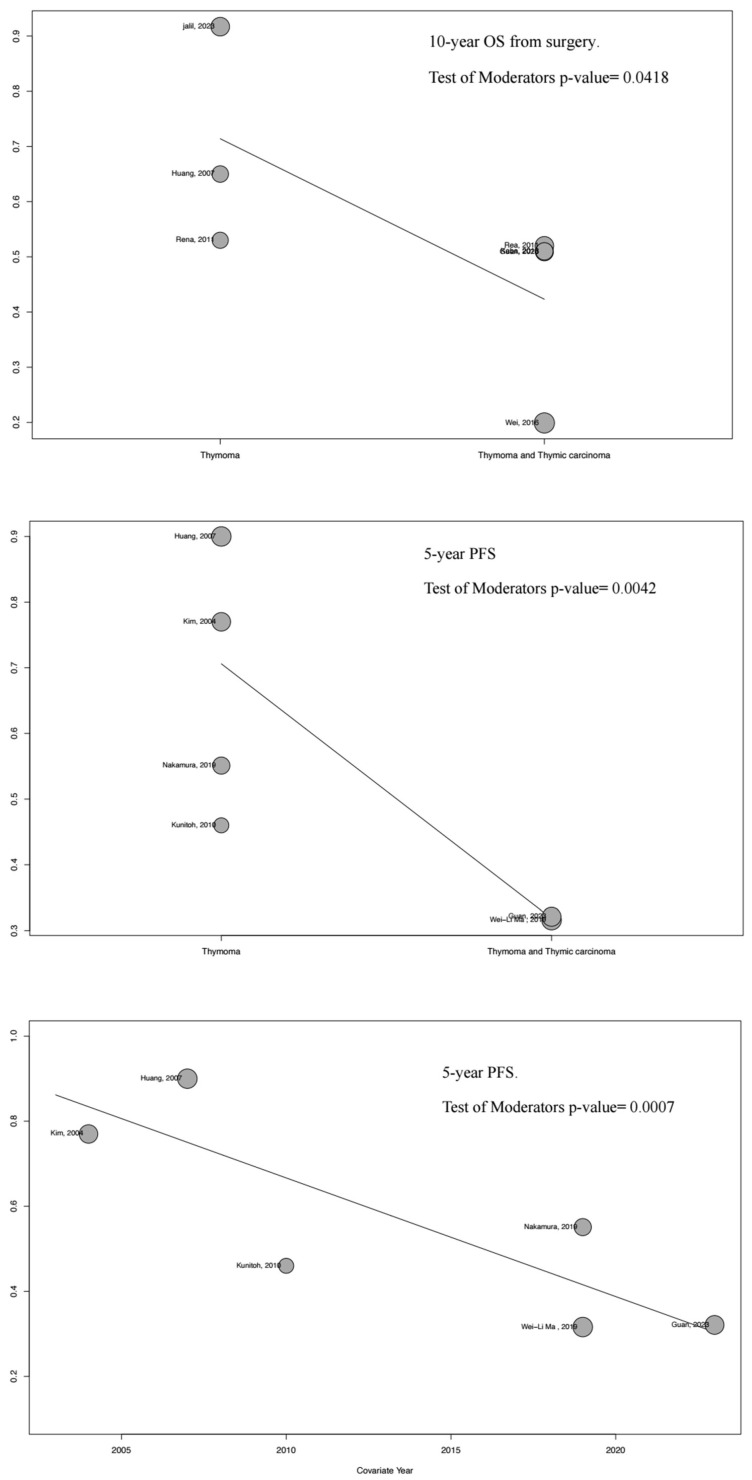
Meta-regression analyses evaluating associations between years of publication, histology and stage with outcomes. We found a significant effect for histology on 10-year OS from surgery and on PFS as well as years of publication on PFS.

We conducted meta-regression analyses evaluating associations between years of publication, histology and stage with outcomes (Fig. 3).

We found no significant effects of factors, except for histology on 10-year OS from surgery (Test of Moderators *p*-value 0.0418) and on PFS (Test of Moderators *p*-value 0.0042) and year on PFS (Test of Moderators *p*-value 0.0007).

### 3.5 Subgroup Analyses

Subgroup analyses were performed to explore the impact of histology and treatment strategy on survival outcomes. When stratified by histology, studies including only thymoma patients showed more favorable survival outcomes compared with those including thymic carcinoma, confirming the known prognostic role of histological subtype. However, the limited number of studies and the heterogeneity of reported data did not allow definitive conclusions. A separate pooled analysis was also performed for studies reporting survival outcomes in patients undergoing upfront surgery. The pooled 5-year OS was 83.5% (95% CI 73.0–93.9), while the pooled 10-year OS was 42.7% (95% CI 14.9–70.5). These results should be interpreted cautiously due to the limited number of studies and the substantial heterogeneity observed. These analyses were exploratory and intended to provide descriptive estimates rather than direct comparisons with induction therapy strategies. Detailed subgroup results are reported in Supplementary Figs. S1 and S2.

### 3.6 Publication Bias

Publication bias analysis was shown in Supplementary Fig. S3. In investigated cases, funnel plot appeared symmetric with asymmetry test not statistically significant, suggesting lack of significant publication bias.

### 3.7 Study Quality

According to the Newcastle–Ottawa Scale, the overall methodological quality of the included studies was moderate. Three studies were classified as high quality (NOS ≥ 8), the majority showed moderate quality (NOS 6–7), and five studies were classified as low quality (NOS ≤ 5). Detailed quality assessment is provided in Supplementary Table S1.

## 4 Discussion

Patients with TETs can present with locally advanced diseases (stage III or IVA), which often pose significant challenges in terms of treatment strategies and prognosis. In such cases, achieving completeness of surgical resection is strongly considered the gold standard to ensure acceptable OS rates and to minimize the risk of recurrence. However, in those patients with tumors initially deemed unresectable due to extensive local invasion or other limiting factors, IT (which may include chemotherapy or chemoradiotherapy), represents a crucial treatment option aimed at downstaging the tumor to enhance subsequent surgical resection.

The effectiveness of IT in increasing the likelihood of complete resection is highly variable, as reported in the literature, with complete resection rates ranging widely between 22% and 92% [23,37]. This variability can be attributed to several factors, including differences in tumor invasiveness, patient selection criteria, and the skill level of the surgeon performing difficult resections. Additionally, advances in imaging techniques and surgical management over the past two decades may have played a role in refining treatment approaches for ATTs.

The present meta-analysis provides a comprehensive assessment of outcomes associated with IT followed by surgery, incorporating data from 24 studies over a 20-year period (2003–2023), with a total of 749 patients analyzed. Our analyses confirm that the 5-yr and 10-yr OS rates after surgical resection remain at 77.5% and 54.3%, respectively. Given the substantial between-study heterogeneity observed for long-term survival outcomes, these pooled estimates should be interpreted as summary indicators of the available literature rather than precise effect measures. Although these findings may reinforce the critical role of multimodal treatment strategies and the expertise of skilled surgical teams in managing these complex malignancies, they should be interpreted as descriptive estimates of outcomes observed in published series rather than as evidence of treatment efficacy, given the observational nature and heterogeneity of the included studies. Nevertheless, with the continuous evolution of neoadjuvant therapy in other cancer types, such as the use of chemo-immunotherapy in non-small cell lung cancer (NSCLC) [38,39,40,41], it is important to assess whether current multimodal strategies are sufficiently effective in modifying prognosis in ATTs. Given the rarity of TETs, meta-analyses in this field often require the inclusion of heterogeneous datasets, and therefore pooled estimates should be interpreted cautiously and considered hypothesis-generating.

In this setting, in our study we performed a rigorous meta-regression analysis to evaluate those factors that influence specific outcomes following IT. Unlike traditional meta-analysis, which synthesizes overall treatment effects, meta-regression enables a deeper exploration of potential sources of heterogeneity among involved studies. Specifically, given the inherent complexity of oncological researches, where patient responses to therapy can vary significantly, meta-regression may help explore potential sources of heterogeneity across studies and provides a tool for suggesting potential associations that should be interpreted cautiously, considering the limited number of studies and the exploratory nature of these analyses [42,43]. Thus, this method allowed us to assess how variables such as treatment period, tumor histology and disease stage impact on OS and PFS.

A major focus in our study was the impact of IT on OS in thymic malignancies, particularly splitting outcomes for both thymoma and thymic carcinoma at 5 and 10 years post-surgery. Previous studies, including recent multicenter real-world analyses of induction therapy outcomes, have reported varying survival results depending on histological subtypes and treatment strategies [44,45]. Compared to previous meta-analyses [46], our results indicate that the 5-yr OS rate remains acceptable at 77.5%, with no statistically significant correlation between OS and treatment year, histology or disease stage. Similarly, the 10-yr OS rate is 54.3%, similar to recent analyses reported by Yang et al. [47], demonstrating very little improvement over the past two decades. These findings underscore an urgent need for novel therapeutic approaches to enhance long-term survival, especially given that, despite advancements in surgical and multimodal management, thymic carcinoma continues to have a poorer prognosis compared to thymoma [48]. In this scenario, future researches should prioritize molecularly-targeted therapies and immunotherapeutic regimens to address these issues in treatment efficacy among different histologies. In addition to OS, our meta-analysis evaluated PFS, which represents another key parameter in determining treatment success. We report a 5-yr PFS of 55.6%, with treatment year and histology emerging as significant influencing factors, whereas disease stage did not show a statistical impact on metaregression. Notably, studies analyzing PFS were performed before 2015 and exclusively focused on thymomas, such as those by Kim and Huang [33,49]. Compared to those authors focusing on both thymoma and thymic carcinoma (published after 2015), these 2 papers demonstrated superior PFS outcomes in thymoma patients, reinforcing the importance of histology as a key prognostic factor for relapse. In this scenario, histology remains the cornerstone for predicting long-term survival in ATTs. As a rule, patients with thymoma generally achieve better treatment outcomes compared to those with thymic carcinoma. The combination of IT and surgery appears to be the most effective strategy for patients with such histology, likely due to its relatively higher chemosensitivity and lower biological aggressiveness [48]. On the contrary, thymic carcinoma is still associated to lower PFS rates, further emphasizing the need for more effective therapeutic strategies to achieve sustained disease stabilization [50]. Furthermore, the optimal IT regimen for thymic carcinoma remains actually undefined [51].

Recent comprehensive reviews further emphasize the need for biomarker-driven therapeutic strategies and improved systemic treatment selection in advanced TETs [52,53]. The absence of genetic profiling in standard clinical practice hinders the ability to personalize therapy based on tumor biology [51]. The development of targeted therapeutic regimens as well as immune checkpoint inhibitors may open the way for improved outcomes. Ongoing trials, such as the phase II Relevent trial [54], evaluating ramucirumab plus carboplatin and paclitaxel in untreated metastatic thymic carcinoma, represent a promising advancement that could reshape the treatment landscape for aggressive thymic tumors, even in the neoadjuvant setting. Within this evolving therapeutic landscape, IT followed by surgery still represents the most commonly adopted multimodal strategy for potentially resectable stage III–IVA TETs in current clinical practice.

Regarding response rates to IT, our analysis indicates a modest but consistent improvement in RR rates, with an observed response rate of 66.8% compared to the 59.2% reported in a previous meta-analysis [46], in line with recent multicenter real-world evidence [44]. Noteworthy, the increased use of advanced imaging techniques, particularly Positron Emission Tomography (PET), may have contributed to more accurate assessments of TETs response [55]. Nonetheless, these improvements in imaging have yet to translate into significant clinical benefits in terms of OS or PFS. Moreover, RR rates were not significantly influenced by treatment year, histology, or disease stage, suggesting a continued reliance on standardized induction protocols (including Platinum, Anthracycline, and Cyclophosphamide regimen) among patients with different histologies as well [56].

Another critical limitation is the heterogeneity in evaluating RR rates after IT across the studies analyzed. Specifically, 11 studies employed the Response Evaluation Criteria In Solid Tumors (RECIST), 4 authors used WHO, while in 4 papers radiological criteria were not reported, leading to potential biases and reducing comparability among studies. To address this limitation, our analysis tried to standardize radiological evaluations wherever possible, further limiting statistical heterogeneity and providing a more accurate depiction of the impact of imaging advancements on treatment assessments. Thus, our findings underscore the need for greater homogeneity in RR criteria in future studies to improve comparability and enhance the accuracy of outcome assessments, as some degree of residual heterogeneity in response assessment remains unavoidable given the variability of reporting across the included studies. As well, combining further meaningful advancement in imaging technologies (in the pre and post-induction setting) along with tailored IT regimens based on tumor biology may lead to significant improvements in long-term survival in such tumors.

## 5 Study Limitations

Regarding the limitations of the included evidence, although the overall methodological quality of the included studies was moderate to high, most data derive from retrospective cohorts, which intrinsically limit causal inference and increase the risk of selection bias and unmeasured confounding. Although study quality was assessed using the Newcastle–Ottawa Scale, the limited number of available studies and the overall moderate-to-high methodological quality did not allow meaningful sensitivity analyses stratified by study quality, and therefore results should be interpreted with this limitation in mind. The relatively small sample sizes of the included studies and the overall quality of the evidence, marked by a significant lack of randomized controlled trials, present substantial challenges; consequently, the overall certainty of evidence should be considered low to very low according to standard methodological frameworks, and findings should be interpreted with appropriate caution. Similarly, findings from meta-regression analyses should be considered exploratory and interpreted cautiously due to the limited number of included studies and the potential risk of spurious associations. While our analysis provides a comprehensive assessment of survival outcomes in a considerable cohort of patients who underwent surgical resection after IT, it is difficult to definitively isolate the independent effects of IT and resection on these outcomes, given the limitations of the available datasets. In addition, a potential survivorship bias should be considered, as the included studies report outcomes only for patients who successfully proceeded to surgical resection after IT, thereby excluding non-responders or patients deemed unsuitable for surgery. Therefore, these findings reflect a selected population of patients able to complete multimodal treatment and may overestimate survival outcomes compared to the entire population receiving IT. Another key limitation of this meta-analysis was our inability to perform separate outcome analyses for thymomas and thymic carcinomas, as many of the included studies reported pooled data for these two histologies. Our exploratory subgroup analyses partially addressed this limitation. Studies including only thymoma patients showed more favorable survival outcomes compared with those including thymic carcinoma, further supporting the well-known prognostic role of histology in advanced TETs. However, these findings should be interpreted cautiously due to the limited number of available studies and the lack of individual patient data. In addition, a separate pooled analysis of studies reporting upfront surgery showed acceptable long-term survival outcomes, although these results represent descriptive estimates and do not allow direct comparisons with multimodality treatment strategies. These findings highlight the need for prospective comparative studies to better define the optimal sequencing between IT and surgery. A direct comparison between patients undergoing IT followed by surgery and those treated with upfront surgery remains particularly challenging in TETs. Due to the rarity of these tumors, treatment allocation is rarely standardized and is usually based on multidisciplinary tumor board evaluation. In clinical practice, patients receiving IT are often those with more locally advanced or borderline resectable disease, whereas patients undergoing upfront surgery are generally considered technically resectable at presentation. This inherent selection bias further limits the validity of indirect comparisons between these treatment strategies. We also reported a significant heterogeneity in PFS, as it was not centrally reviewed in most of these trials and, especially in thymomas, local progressions were sometimes not considered a “true” progressive disease. Another methodological limitation relates to the heterogeneity in survival time origin across studies, as OS was variably calculated from the start of induction therapy or from the time of surgery: these definitions are not equivalent and may introduce immortal time bias; therefore, survival estimates should be interpreted cautiously when comparing studies using different time origins. Formal sensitivity analyses were not performed due to the limited number of available studies and the small proportion of high-quality studies (3 studies); however, restricting the primary analyses to studies reporting survival from a uniform time origin (surgery) represents a methodological approach aimed at improving comparability and partially mitigating this source of heterogeneity. Despite the high heterogeneity observed across several outcomes, pooled estimates were considered appropriate given the exploratory nature of this meta-analysis and the rarity of TETs, which limits the feasibility of large homogeneous cohorts. Random-effects models were consistently applied to account for between-study variability and to provide more conservative estimates. Accordingly, pooled estimates should be regarded as descriptive summary measures rather than stable effect estimates applicable across all clinical settings. Moreover, recurrence rates following surgical resection of TETs remain a concern, often emerging many years after surgery. As a result, the follow-up durations in the included studies were relatively short, with median follow-up periods ranging from 27 to 94 months. Finally, the data available from our meta-analysis were insufficient to determine which specific chemotherapy regimens are most suitable for IT by histology. This limitation is mainly due to the substantial heterogeneity in IT strategies across studies, including differences in chemotherapy protocols, the number of cycles, and the use of radiotherapy. Due to the limited and inconsistently reported data, meaningful subgroup analyses according to specific induction regimens (e.g., chemotherapy versus chemoradiotherapy) were not feasible, and this should be considered when interpreting the clinical applicability of pooled estimates. In addition, some limitations related to the review process itself should be acknowledged. Despite adherence to PRISMA guidelines, the inclusion of mainly retrospective studies, the potential for publication bias (which cannot be definitively excluded given the limited number of studies), and the lack of individual patient data may have influenced the robustness of pooled estimates. Moreover, the rarity of TETs limits the availability of large homogeneous datasets.

## 6 Conclusions

Our meta-analysis suggests that IT followed by surgical resection is associated with promising long-term outcomes in patients with ATTs. These findings support the use of IT within multimodal treatment strategies for selected patients with initially unresectable or borderline resectable stage III–IVA TETs, although the available evidence remains observational and does not allow definitive conclusions regarding its independent impact on survival. From a clinical perspective, IT should be considered particularly in patients with ATTs in whom complete resection is considered uncertain at diagnosis, whereas upfront surgery remains the preferred strategy for clearly resectable tumors. These findings further emphasize the importance of multidisciplinary evaluation in experienced centers to optimize treatment sequencing. However, given the complexity of treatment decision-making in ATTs and the relative absence of tailored chemotherapeutic regimens (even after 20 years of multimodal oncological trials), further multicenter prospective studies are warranted to better define which subgroup of patients may benefit most from IT, to evaluate the impact of different chemo- and immunotherapeutic agents on tumor response and downstaging, and to refine preoperative assessment strategies in order to optimize treatment selection and enhance patient outcomes.

## Data Availability

The authors confirm that the data supporting the findings of this study are available within the article and its Supplementary Materials.

## Supplementary Materials

The supplementary material is available online at https://www.techscience.com/doi/10.32604/or.2026.077158/s1.

## Abbreviations

TETs	Thymic epithelial tumors
ATTs	Advanced thymic tumors
IT	Induction therapy
RR	Radiological response
PFS	Progression-free survival
OS	Overall survival
PR	Partial response
CR	Complete response
PET	Positron emission tomography
RECIST	Response Evaluation Criteria in Solid Tumors
WHO	World Health Organization
NOS	Newcastle–Ottawa Scale
CI	Confidence interval
SE	Standard error
NSCLC	Non-small cell lung cancer
PRISMA	Preferred Reporting Items for Systematic Reviews and Meta-Analyses
PROSPERO	Prospective Register of Systematic Reviews

## Appendix A Detailed Search Strategy

**PubMed (MEDLINE)**

(“thymus neoplasms” [MeSH Terms] OR “thymoma” [Title/Abstract] OR “thymic carcinoma” [Title/Abstract]) AND (“neoadjuvant therapy” [MeSH Terms] OR “induction therapy” [Title/Abstract]) AND (“surgery” [Title/Abstract] OR “surgical resection” [Title/Abstract]) AND (“survival” [Title/Abstract] OR “overall survival” [Title/Abstract] OR “progression-free survival” [Title/Abstract]).

**Embase**

(‘thymus tumor’/exp OR ‘thymoma’: ti,ab OR ‘thymic carcinoma’: ti,ab) AND (‘neoadjuvant therapy’/exp OR ‘induction therapy’: ti,ab) AND (‘surgery’/exp OR ‘surgical resection’: ti,ab) AND (‘survival’/exp OR ‘overall survival’: ti,ab OR ‘progression free survival’: ti,ab).

**Cochrane Central Register of Controlled Trials (CENTRAL)**

(thymoma OR “thymic carcinoma” OR “thymus neoplasm”) AND (“neoadjuvant therapy” OR “induction therapy”) AND (surgery OR “surgical resection”) AND (survival OR “overall survival” OR “progression-free survival”).
