# Supplementary material for: Induction Therapy Followed by Surgery in Advanced Thymic Epithelial Tumors: A 20-Year Systematic Review and Meta-Analysis
Source: Oncol Res. 2026 Jul 16;34(8):2. doi: 10.32604/or.2026.077158 (PMC13397335; doi:10.32604/or.2026.077158)
Supplement: Supplementary file 1 [file OncolRes-34-77158-s001.zip › OR-77158-Supplementary_Files-123365-2.docx]

| **Section and Topic** | **Item No** | **Checklist item** | **Reported on Page Number/Line Number** | **Reported on Section/Paragraph** |
| --- | --- | --- | --- | --- |
| **TITLE** | | |  |  |
| Title | 1 | Identify the report as a systematic review. | Page 1 / Lines 3-5 | Title (Systematic Review and Meta-analysis) |
| **ABSTRACT** | | |  |  |
| Abstract | 2 | See the PRISMA 2020 for Abstracts checklist. | Page 1 / Lines 18-38 | Abstract (structured abstract compliant with PRISMA 2020 for Abstracts) |
| **INTRODUCTION** | | |  |  |
| Rationale | 3 | Describe the rationale for the review in the context of existing knowledge. | Page 4 / Lines 72–95 | Introduction, paragraph 1–2 |
| Objectives | 4 | Provide an explicit statement of the objective(s) or question(s) the review addresses. | Page 4 / Lines 96-100 | Introduction, final paragraph |
| **METHODS** | | |  |  |
| Eligibility criteria | 5 | Specify the inclusion and exclusion criteria for the review and how studies were grouped for the syntheses. | Page 5 / Lines 114–122 | Methods – Study selection / Eligibility criteria |
| Information sources | 6 | Specify all databases, registers, websites, organisations, reference lists and other sources searched or consulted to identify studies. Specify the date when each source was last searched or consulted. | Page 5 / Lines 104–107 | Methods – Search strategy |
| Search strategy | 7 | Present the full search strategies for all databases, registers and websites, including any filters and limits used. | Page 5 / Lines 104–113 | Methods – Search strategy - Figure 1 - Supplementary Appendix 1 |
| Selection process | 8 | Specify the methods used to decide whether a study met the inclusion criteria of the review, including how many reviewers screened each record and each report retrieved, whether they worked independently, and if applicable, details of automation tools used in the process. | Page 5 / Lines 109–113 | Methods – Study selection |
| Data collection process | 9 | Specify the methods used to collect data from reports, including how many reviewers collected data from each report, whether they worked independently, any processes for obtaining or confirming data from study investigators, and if applicable, details of automation tools used in the process. | Page 6 / Lines 126–130 | Methods – Data extraction |
| Data items | 10a | List and define all outcomes for which data were sought. Specify whether all results that were compatible with each outcome domain in each study were sought (e.g. for all measures, time points, analyses), and if not, the methods used to decide which results to collect. | Page 6 / Lines 132–137 | Methods – Outcomes definition |
|  | 10b | List and define all other variables for which data were sought (e.g. participant and intervention characteristics, funding sources). Describe any assumptions made about any missing or unclear information. | Page 6 / Lines 126–130 | Methods – Data extraction / Study characteristics |
| Study risk of bias assessment | 11 | Specify the methods used to assess risk of bias in the included studies, including details of the tool(s) used, how many reviewers assessed each study and whether they worked independently, and if applicable, details of automation tools used in the process. | Page 6 / Lines 123–125 | Methods – Quality assessment |
| Effect measures | 12 | Specify for each outcome the effect measure(s) (e.g. risk ratio, mean difference) used in the synthesis or presentation of results. | Page 6 / Lines 132–137 | Methods – Statistical analysis |
| Synthesis methods | 13a | Describe the processes used to decide which studies were eligible for each synthesis (e.g. tabulating the study intervention characteristics and comparing against the planned groups for each synthesis (item #5)). | Page 6 / Lines 139–146 | Methods – Statistical analysis |
|  | 13b | Describe any methods required to prepare the data for presentation or synthesis, such as handling of missing summary statistics, or data conversions. | Page 6 / Lines 139–146 | Methods – Statistical analysis |
|  | 13c | Describe any methods used to tabulate or visually display results of individual studies and syntheses. | Page 6 / Lines 140–143 | Methods – Statistical analysis |
|  | 13d | Describe any methods used to synthesize results and provide a rationale for the choice(s). If meta-analysis was performed, describe the model(s), method(s) to identify the presence and extent of statistical heterogeneity, and software package(s) used. | Page 6 / Lines 144–149 | Methods – Statistical analysis |
|  | 13e | Describe any methods used to explore possible causes of heterogeneity among study results (e.g. subgroup analysis, meta-regression). | Page 6 / Lines 144–149 | Methods – Statistical analysis (Subgroup analyses) |
|  | 13f | Describe any sensitivity analyses conducted to assess robustness of the synthesized results. | Page 6 / Lines 144–150 | Methods – Statistical analysis |
| Reporting bias assessment | 14 | Describe any methods used to assess risk of bias due to missing results in a synthesis (arising from reporting biases). | Page 6 / Lines 142–143 | Methods – Statistical analysis (publication bias assessment) |
| Certainty assessment | 15 | Describe any methods used to assess certainty (or confidence) in the body of evidence for an outcome. | Not performed | Not performed |
| **RESULTS** | | |  |  |
| Study selection | 16a | Describe the results of the search and selection process, from the number of records identified in the search to the number of studies included in the review, ideally using a flow diagram. | Page 7 / Lines 155–159 + Figure 1 | Results – Study selection Supplementary Figure 1 (PRISMA flow diagram) |
|  | 16b | Cite studies that might appear to meet the inclusion criteria, but which were excluded, and explain why they were excluded. | Not applicable (no reports excluded after full-text assessment) | Results – Study selection |
| Study characteristics | 17 | Cite each included study and present its characteristics. | Page 7–10 / Lines 165–250 + Tables 1–3 | Results – Study characteristics (Table 1-3) |
| Risk of bias in studies | 18 | Present assessments of risk of bias for each included study. | Page 11 / Lines 308–312 | Results – Study quality Supplementary Table 1 |
| Results of individual studies | 19 | For all outcomes, present, for each study: (a) summary statistics for each group (where appropriate) and (b) an effect estimate and its precision (e.g. confidence/credible interval), ideally using structured tables or plots. | Page 8–10 / Tables 2–3 | Results – Primary outcomes Figures 2–4 (forest plots) |
| Results of syntheses | 20a | For each synthesis, briefly summarise the characteristics and risk of bias among contributing studies. | Page 8 / Lines 179–185 | Results – Primary outcomes |
|  | 20b | Present results of all statistical syntheses conducted. If meta-analysis was done, present for each the summary estimate and its precision (e.g. confidence/credible interval) and measures of statistical heterogeneity. If comparing groups, describe the direction of the effect. | Page 10–11 / Lines 226–278 + Figure 2 | Results – Primary outcomes + Figure 2 |
|  | 20c | Present results of all investigations of possible causes of heterogeneity among study results. | Page 11 / Lines 281–287 + Figure 3 | Results – Primary outcomes + Figure 3 |
|  | 20d | Present results of all sensitivity analyses conducted to assess the robustness of the synthesized results. | Not performed | Not performed (no sensitivity analyses planned) |
| Reporting biases | 21 | Present assessments of risk of bias due to missing results (arising from reporting biases) for each synthesis assessed. | Page 11 / Lines 304–307 | Results – Publication bias analysis |
| Certainty of evidence | 22 | Present assessments of certainty (or confidence) in the body of evidence for each outcome assessed. | Not assessed | Not assessed |
| **DISCUSSION** | | |  |  |
| Discussion | 23a | Provide a general interpretation of the results in the context of other evidence. | Page 12 / Lines 315–339 | Discussion – first paragraph |
|  | 23b | Discuss any limitations of the evidence included in the review. | Page 13 / Lines 387–416 | Discussion – Limitations of included studies |
|  | 23c | Discuss any limitations of the review processes used. | Page 13 / Lines 417–423 | Discussion – Study limitations |
|  | 23d | Discuss implications of the results for practice, policy, and future research. | Page 13–14 / Lines 424–434 | Discussion – Clinical implications and future research |
| **OTHER INFORMATION** | | |  |  |
| Registration and protocol | 24a | Provide registration information for the review, including register name and registration number, or state that the review was not registered. | Page 5 / Lines 101–104 | Methods – Registration: PROSPERO ID: CRD420251026044 |
|  | 24b | Indicate where the review protocol can be accessed, or state that a protocol was not prepared. | Not applicable | Not applicable |
|  | 24c | Describe and explain any amendments to information provided at registration or in the protocol. | Not applicable | Not applicable |
| Support | 25 | Describe sources of financial or non-financial support for the review, and the role of the funders or sponsors in the review. | Page 14 / Lines 446–448 | Funding statement |
| Competing interests | 26 | Declare any competing interests of review authors. | Page 14 / Lines 464–467 | Conflict of Interest statement |
| Availability of data, code and other materials | 27 | Report which of the following are publicly available and where they can be found: template data collection forms; data extracted from included studies; data used for all analyses; analytic code; any other materials used in the review. | Page 14 / Lines 457–459 | Data availability statement |

*As the checklist was provided upon initial submission, the page number/line number reported may be changed due to copyediting and may not be referable in the published version. In this case, the section/paragraph may be used as an alternative reference.
