# Supplementary material for: Induction Therapy Followed by Surgery in Advanced Thymic Epithelial Tumors: A 20-Year Systematic Review and Meta-Analysis
Source: Oncol Res. 2026 Jul 16;34(8):2. doi: 10.32604/or.2026.077158 (PMC13397335; doi:10.32604/or.2026.077158)
Supplement: Supplementary file 1 [file OncolRes-34-77158-s001.zip › OR-77158-Supplementary_Files.docx]

**Supplementary Table S1:** Methodological quality assessment of included studies using the Newcastle–Ottawa Scale (NOS).

| **Study** | **Year** | **Selection** | **Comparability** | **Outcome** | **Total** |
| --- | --- | --- | --- | --- | --- |
| Venuta [16] | 2003 | 3 | 1 | 2 | **6** |
| Kim [9] | 2004 | 3 | 1 | 2 | **6** |
| Lucchi [20] | 2006 | 3 | 1 | 2 | **6** |
| Huang [19] | 2007 | 3 | 1 | 2 | **6** |
| Wright [26] | 2008 | 2 | 1 | 2 | **5** |
| Mineo [17] | 2010 | 3 | 1 | 2 | **6** |
| Cardillo [25] | 2010 | 3 | 1 | 2 | **6** |
| Kunitoh [7] | 2010 | 3 | 1 | 2 | **6** |
| Rena [18] | 2011 | 3 | 1 | 2 | **6** |
| Rea [24] | 2011 | 3 | 1 | 2 | **6** |
| Park [23] | 2013 | 3 | 1 | 2 | **6** |
| Filosso [28] | 2013 | 2 | 1 | 2 | **5** |
| Korst [10] | 2014 | 3 | 1 | 2 | **6** |
| Shintani [34] | 2014 | 2 | 1 | 2 | **5** |
| Cardillo [27] | 2015 | 4 | 2 | 3 | **9** |
| Leuzzi [31] | 2015 | 3 | 2 | 3 | **8** |
| Wei [36] | 2016 | 4 | 2 | 3 | **9** |
| Kaba [30] | 2018 | 2 | 1 | 2 | **5** |
| Park [33] | 2018 | 3 | 1 | 2 | **6** |
| Ma [32] | 2019 | 3 | 1 | 2 | **6** |
| Nakamura [22] | 2019 | 3 | 1 | 2 | **6** |
| Suh [35] | 2019 | 3 | 1 | 2 | **6** |
| Guan [29] | 2023 | 3 | 1 | 2 | **6** |
| Abdel Jalil [21] | 2023 | 2 | 1 | 2 | **5** |


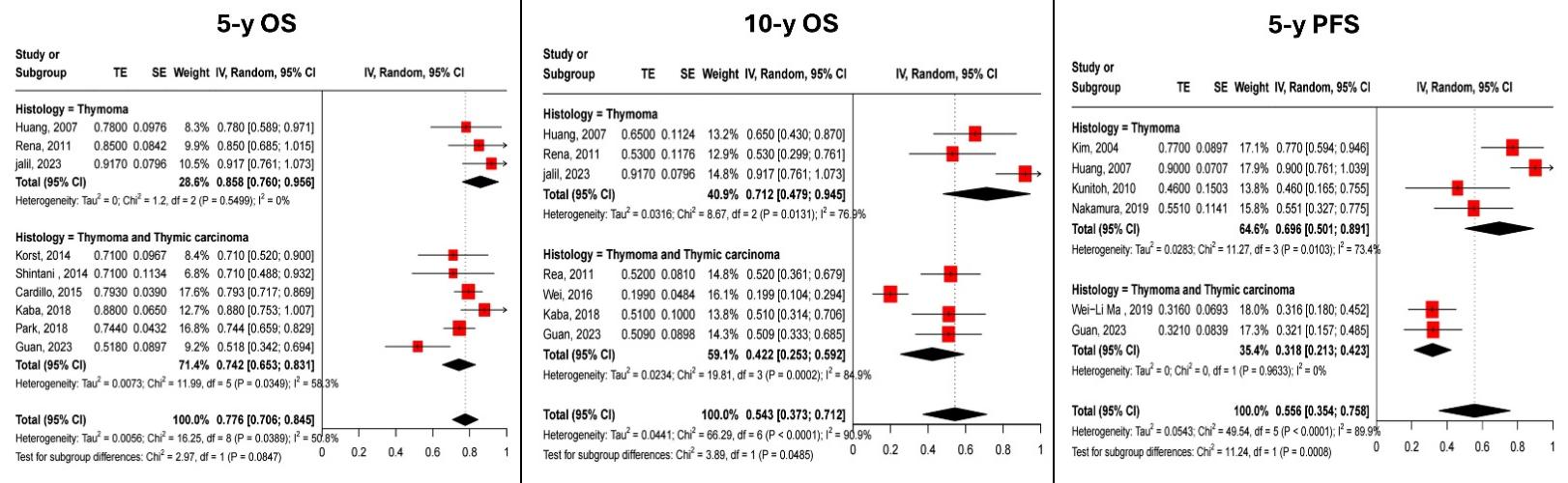


**Supplementary Figure S1:** Subgroup analyses according to histology. OS: overall survival; PFS: progression-free survival; CI: confidence interval; TE: treatment effect; SE: standard error; IV: inverse variance method; Tau²: between-study var-iance; Chi²: Cochran’s Q statistic; I²: inconsistency index.


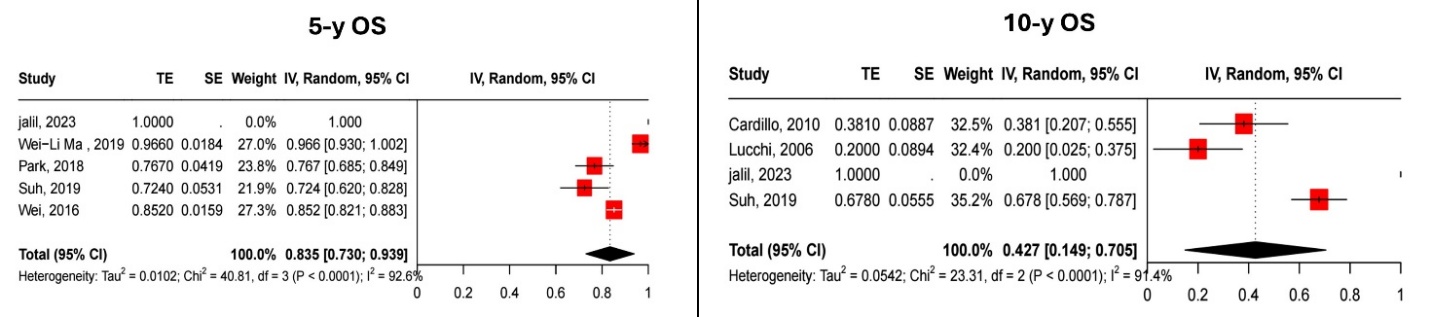


**Supplementary Figure S2:** Pooled analysis of overall survival in studies reporting upfront surgery. OS: overall survival. CI: confidence interval; TE: treatment effect; SE: standard error; IV: inverse variance method; Tau²: between-study var-iance; Chi²: Cochran’s Q statistic; I²: inconsistency index.


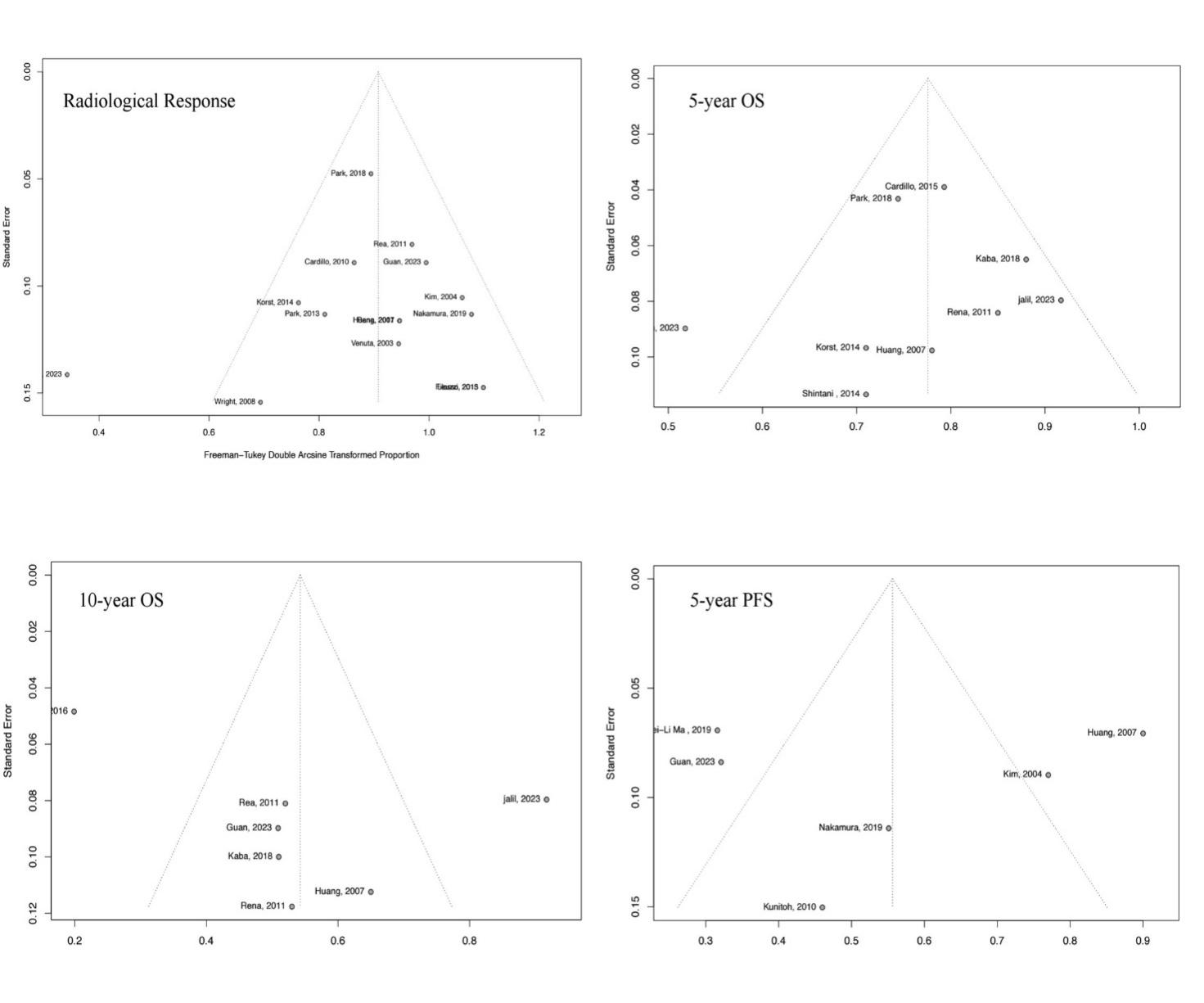


**Supplementary Figure S3:** Publication bias analyses on reported outcomes. OS: overall survival. PFS: progression-free survival.

References

[7] Kunitoh H, Tamura T, Shibata T, Takeda K, Katakami N, Nakagawa K, et al. A phase II trial of dose-dense chemotherapy, followed by surgical resection and/or thoracic radiotherapy, in locally advanced thymoma: report of a Japan Clinical Oncology Group trial (JCOG 9606). Br J Cancer. 2010;103(1):6–11. doi:10.1038/sj.bjc.6605731.

[9] Kim ES, Putnam JB, Komaki R, Walsh GL, Ro JY, Shin HJ, et al. Phase II study of a multidisciplinary approach with induction chemotherapy, followed by surgical resection, radiation therapy, and consolidation chemotherapy for unresectable malignant thymomas: final report. Lung Cancer. 2004;44(3):369–79. doi:10.1016/j.lungcan.2003.12.010.

[10] Korst RJ, Bezjak A, Blackmon S, Choi N, Fidias P, Liu G, et al. Neoadjuvant chemoradiotherapy for locally advanced thymic tumors: a phase II, multi-institutional clinical trial. J Thorac Cardiovasc Surg. 2014;147(1):36–46.e1. doi:10.1016/j.jtcvs.2013.08.061.

[16] Venuta F, Rendina EA, Longo F, De Giacomo T, Anile M, Mercadante E, et al. Long-term outcome after multimodality treatment for stage III thymic tumors. Ann Thorac Surg. 2003;76(6):1866–72. doi:10.1016/S0003-4975(03)01020-8.

[17] Mineo TC, Mineo D, Onorati I, Cufari ME, Ambrogi V. New predictors of response to neoadjuvant chemotherapy and survival for invasive thymoma: a retrospective analysis. Ann Surg Oncol. 2010;17(11):3022–9. doi:10.1245/s10434-010-1134-9.

[18] Rena O, Mineo TC, Casadio C. Multimodal treatment for stage IVA thymoma: a proposable strategy. Lung Cancer. 2012;76(1):89–92. doi:10.1016/j.lungcan.2011.10.004.

[19] Huang J, Rizk NP, Travis WD, Seshan VE, Bains MS, Dycoco J, et al. Feasibility of multimodality therapy including extended resections in stage IVA thymoma. J Thorac Cardiovasc Surg. 2007;134(6):1477–84. doi:10.1016/j.jtcvs.2007.07.049.

[20] Lucchi M, Melfi F, Dini P, Basolo F, Viti A, Givigliano F, et al. Neoadjuvant chemotherapy for stage III and IVA thymomas: a single-institution experience with a long follow-up. J Thorac Oncol. 2006;1(4):308–13. doi:10.1016/S1556-0864(15)31586-0.

[21] Abdel Jalil R, Abdallah FA, Obeid Z, Harb AK, Abou Chaar MK, Shannies TB, et al. Locally advanced thymoma; does neoadjuvant chemotherapy make a difference? J Cardiothorac Surg. 2023;18(1):245. doi:10.1186/s13019-023-02357-4.

[22] Nakamura S, Kawaguchi K, Fukui T, Hakiri S, Ozeki N, Mori S, et al. Multimodality therapy for thymoma patients with pleural dissemination. Gen Thorac Cardiovasc Surg. 2019;67(6):524–9. doi:10.1007/s11748-018-01054-7.

[23] Park S, Ahn MJ, Ahn JS, Sun JM, Shim YM, Kim J, et al. A prospective phase II trial of induction chemotherapy with docetaxel/cisplatin for masaoka stage III/IV thymic epithelial tumors. J Thorac Oncol. 2013;8(7):959–66. doi:10.1097/JTO.0b013e318292c41e.

[24] Rea F, Marulli G, di Chiara F, Schiavon M, Perissinotto E, Breda C, et al. Multidisciplinary approach for advanced stage thymic tumors: long-term outcome. Lung Cancer. 2011;72(1):68–72. doi:10.1016/j.lungcan.2010.07.006.

[25] Cardillo G, Carleo F, Giunti R, Lopergolo MG, Salvadori L, De Massimi AR, et al. Predictors of survival in patients with locally advanced thymoma and thymic carcinoma (Masaoka stages III and IVa). Eur J Cardiothorac Surg. 2010;37(4):819–23. doi:10.1016/j.ejcts.2009.11.001.

[26] Wright CD, Choi NC, Wain JC, Mathisen DJ, Lynch TJ, Fidias P. Induction chemoradiotherapy followed by resection for locally advanced masaoka stage III and IVA thymic tumors. Ann Thorac Surg. 2008;85(2):385–9. doi:10.1016/j.athoracsur.2007.08.051.

[27] Cardillo G, Lucchi M, Marulli G, Infante M, Leuzzi G, Mussi A, et al. Induction therapy followed by surgical resection in Stage-III thimic epithelial tumors: long-term results from a multicentre analysis of 108 cases. Lung Cancer. 2016;93:88–94. doi:10.1016/j.lungcan.2016.01.008.

[28] Filosso PL, Guerrera F, Rendina AE, Bora G, Ruffini E, Novero D, et al. Outcome of surgically resected thymic carcinoma: a multicenter experience. Lung Cancer. 2014;83(2):205–10. doi:10.1016/j.lungcan.2013.11.015.

[29] Guan S, Long W, Liu Y, Cai B, Luo J. Prognosis of concurrent versus sequential chemo-radiotherapy induction followed by surgical resection in patients with advanced thymic epithelial tumors: a retrospective study. Ann Surg Oncol. 2023;30(11):6739–47. doi:10.1245/s10434-023-13954-x.

[30] Kaba E, Ozkan B, Erus S, Duman S, Cimenoglu B, Toker A. Role of surgery in the treatment of masaoka stage IVa thymoma. Ann Thorac Cardiovasc Surg. 2018;24(1):6–12. doi:10.5761/atcs.oa.17-00108.

[31] Leuzzi G, Alessandrini G, Sperduti I, Forcella D, Marino M, Ceribelli A, et al. Induction therapy versus initial surgery in advanced thymic tumors: perioperative and oncological outcome. Thorac Cardiovasc Surg. 2017;65(3):234–43. doi:10.1055/s-0035-1564890.

[32] Ma WL, Lin CC, Hsu FM, Lee JM, Chen JS, Hsieh MS, et al. Clinical outcomes of up-front surgery versus surgery after induction chemotherapy for thymoma and thymic carcinoma: a retrospective study. Clin Lung Cancer. 2019;20(6):e609–18. doi:10.1016/j.cllc.2019.06.011.

[33] Park S, Park IK, Kim YT, Lee GD, Kim DK, Cho JH, et al. Comparison of neoadjuvant chemotherapy followed by surgery to upfront surgery for thymic malignancy. Ann Thorac Surg. 2019;107(2):355–62. doi:10.1016/j.athoracsur.2018.08.055.

[34] Shintani Y, Inoue M, Kawamura T, Funaki S, Minami M, Okumura M. Multimodality treatment for advanced thymic carcinoma: outcomes of induction therapy followed by surgical resection in 16 cases at a single institution. Gen Thorac Cardiovasc Surg. 2015;63(3):159–63. doi:10.1007/s11748-014-0486-7.

[35] Suh JW, Park SY, Lee CY, Song SH, Kim DJ, Paik HC, et al. Neoadjuvant therapy for thymic neoplasms reduces tumor volume per 3D-reconstructed images but does not improve the complete resection rate. PLoS One. 2019;14(3):e0214291. doi:10.1371/journal.pone.0214291.

[36] Wei Y, Gu Z, Shen Y, Fu J, Tan L, Zhang P, et al. Preoperative induction therapy for locally advanced thymic tumors: a retrospective analysis using the ChART database. J Thorac Dis. 2016;8(4):665–72. doi:10.21037/jtd.2016.03.02.
